# Inhibition of Breast Cancer Resistance Protein (ABCG2) in Human Myeloid Dendritic Cells Induces Potent Tolerogenic Functions during LPS Stimulation

**DOI:** 10.1371/journal.pone.0104753

**Published:** 2014-08-11

**Authors:** Jun-O Jin, Wei Zhang, Ka-Wing Wong, Minseok Kwak, Ian R. van Driel, Qing Yu

**Affiliations:** 1 Shanghai Public Health Clinical Center, Shanghai Medical College, Fudan University, Shanghai, China; 2 Department of Chemistry, Pukyong National University, Busan, South Korea; 3 Department of Biochemistry and Molecular Biology, Bio21 Molecular Science and Biotechnology Institute, University of Melbourne, Melbourne, Victoria, Australia; 4 Department of Immunology and Infectious Diseases, The Forsyth Institute, Cambridge, Massachusetts, United States of America; George Mason University, United States of America

## Abstract

Breast cancer resistance protein (ABCG2), a member of the ATP-binding cassette transporters has been identified as a major determinant of multidrug resistance (MDR) in cancer cells, but ABC transporter inhibition has limited therapeutic value *in vivo*. In this research, we demonstrated that inhibition of efflux transporters ABCG2 induced the generation of tolerogenic DCs from human peripheral blood myeloid DCs (mDCs). ABCG2 expression was present in mDCs and was further increased by LPS stimulation. Treatment of CD1c^+^ mDCs with an ABCG2 inhibitor, Ko143, during LPS stimulation caused increased production of IL-10 and decreased production of pro-inflammatory cytokines and decreased expression of CD83 and CD86. Moreover, inhibition of ABCG2 in monocyte-derived DCs (MDDCs) abrogated the up-regulation of co-stimulatory molecules and production of pro-inflammatory cytokines in these cells in response to LPS. Furthermore, CD1c^+^ mDCs stimulated with LPS plus Ko143 inhibited the proliferation of allogeneic and superantigen-specific syngenic CD4^+^ T cells and promoted expansion of CD25^+^FOXP3^+^ regulatory T (Treg) cells in an IL-10-dependent fashion. These tolerogenic effects of ABCG2 inhibition could be abolished by ERK inhibition. Thus, we demonstrated that inhibition of ABCG2 in LPS-stimulated mDCs can potently induce tolerogenic potentials in these cells, providing crucial new information that could lead to development of better strategies to combat MDR cancer.

## Introduction

Multidrug resistance (MDR) in cancer cells is the primary cause of cancer chemotherapy failure. One of the major determinants of the MDR phenotype is the over-expression of ATP-binding cassette (ABC) transporters, which reduces the intracellular accumulation of an anti-cancer drug [1]. An increasing amount of evidence is showing that in addition to contributing to drug resistance, ABC transporters play an important role in genesis of tumor [2]. Some reports have attributed the failure of chemotherapy to the survival of multidrug chemo-resistant cancer stem/initiating cells [3], [4]. High levels of breast cancer resistance protein (ABCG2) gene are expressed in the surface membrane of MDR cancer cells, where it transports anti-cancer drugs including doxorubicin, mitoxantrone, topotecan, and daunorubicin [3], [17]. Therefore, much effort has been devoted to developing ABC transporter inhibitors such as ABCB1 and ABCG2 in the hope that they would kill drug resistance cancer cells [5], [6]. Although these ABC transporter inhibitors have shown promising activities in preclinical studies and early clinical trials as effective and nontoxic inhibitors, most of them have been found to lack significant efficacy in late-phase clinical trials [7]–[9].

Dendritic cells (DCs) are the most efficient antigen-presenting cells (APCs) and the essential components of cancer vaccine through their capacity to capture, process, and present antigens to T cells [10]. Maturation and activation of DCs are pivotal events in the control of innate and adaptive immunity [11]. In addition to activation of adaptive immune responses, DCs also play an important role in induction of immune tolerance [12], [13]. Tolerogenic DCs are characterized by a semi-mature phenotype, with low levels of co-stimulatory molecule expression [14], [15]. Moreover, tolerogenic DCs produce high levels of anti-inflammatory cytokines and low levels of pro-inflammatory cytokines, resulting in the induction and expansion of regulatory T (Treg) cells [12],[16].

Recently, a number of investigations have been performed to examine the function of ABC transporters in immune modulation. Interestingly, ABCG2 is also expressed on CD34^+^ hematopoietic stem cells, monocyte-derived DCs (MDDCs) and skin langerhans cells (LCs) and plays a roles in the differentiation and migration of these cells [18]–[20]. Moreover, peroxisome proliferator-activated receptor-γ (PPAR-γ) stimulation in MDDCs promotes up-regulation of ABCG2 expression and enhances their capacity to extrude xenobiotics [21]. However, the function of ABCG2 in the maturation of DCs, especially human blood myeloid DCs (mDCs), is not well-characterized. In this study, we found that human blood CD1c^+^ mDCs express functional ABCG2 on cell surface and the expression of this molecule is further enhanced by lipopolysaccharide (LPS). Moreover, we identified ABCG2 as a critical factor required for the maturation of DCs and inhibition of ABCG2 during LPS-induced DC maturation promotes the generation of immune tolerogenic DCs as a new immune-regulatory mechanism. This finding may provide insights for improving therapeutic strategies for MDR cancer.

## Results

### LPS induces up-regulation of ABCG2 expression in PBDCs

**Figure 1:**
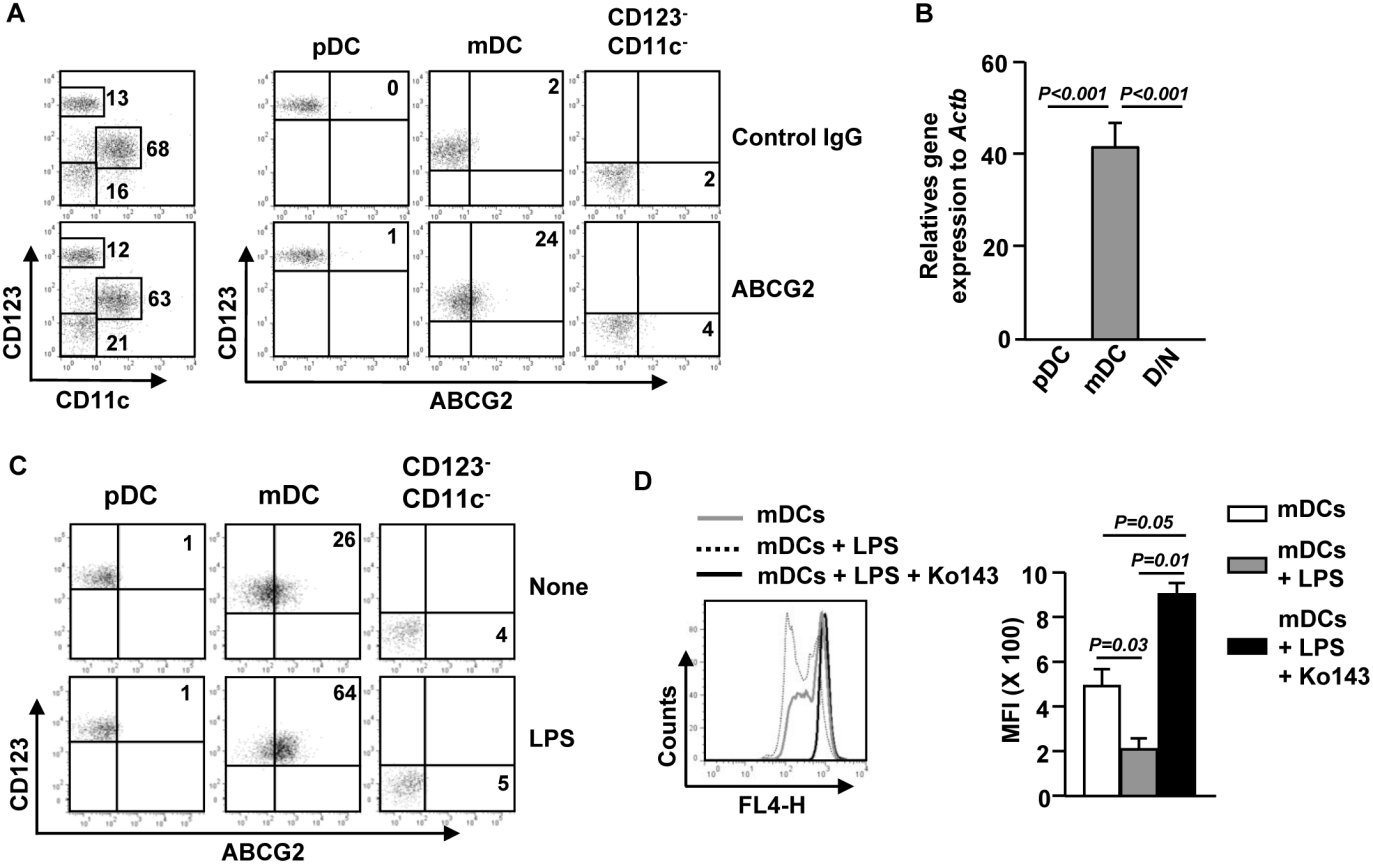
LPS induces over-expression of ABCG2 in blood mDCs. (A) PBDCs were purified from peripheral blood by PBDC isolation kit. Surface ABCG2 expression levels were measured in CD11c^−^CD123^+^ pDCs, CD11c^+^CD123^inter^ mDCs and CD11c^−^CD123^−^ cells by flow cytometry. (B) Real-time PCR analysis of ABCG2 gene expression, presented relative to that of β-actin, in purified pDCs, mDCs and CD11c^−^CD123^−^ cells (D/N). Data are representative or the average of analyses of 4 samples from 4 donors for each group. (C) PBDCs were stimulated with LPS for 24 hours. ABCG2 expression in gated pDCs, mDCs and CD11c^−^CD123^−^ cells were analyzed on a flow cytometry. Data are representative of analyses of 3 samples from 3 donors. (D) Mitoxantrone efflux in purified mDCs and LPS-stimulated mDCs in the presence or absence of Ko143 was analyzed by flow cytometry (left panel) and mean fluorescence intensity (MFI) was shown (right panel). Data are representative or the average of analyses of 3 samples from 3 donors.

Previous reports have shown that *ex vivo* human MDDCs and LCs express ABCG2 on their surface, but the expression of ABCG2 in human blood DC subsets has not been investigated. We therefore assessed the expression levels of ABCG2 in human peripheral blood CD11c^+^CD123^inter^ mDCs and CD11c^−^CD123^+^ plasmacytoid DCs (pDCs). mDCs, but not pDCs, expressed detectable levels of ABCG2 on cell surface (Figure 1A). To confirm this observation, we sorted PBDCs into 3 populations: CD11c^−^CD123^+^ pDCs, CD11c^+^CD123^inter^ mDCs and CD11c^−^CD123^−^ cells, which are non-DCs, and measured mRNA levels of ABCG2 mRNA. As expected, only the mDC population expressed high levels of ABCG2 mRNA (Figure 1B). Since DCs can alter the expression of some surface proteins during activation and maturation, we next assessed whether LPS stimulation can alter ABCG2 expression in MDDCs and PBDCs. Interestingly, LPS stimulation induced up-regulation of ABCG2 expression in MDDCs (Figure S1A) and blood mDCs, but not in pDCs (Figure 1C). Consistent with the changes at protein levels, LPS treatment led to marked increases in ABCG2 mRNA levels in MDDCs (Figure S1B).

Next we assessed the effect of an ABCG2 inhibitor on the mitoxantrone efflux capacity of mDCs, with or without LPS stimulation, to determine whether ABCG2 expressed by mDCs is functional. LPS-stimulated mDCs showed a marked decrease in mitoxantrone labeling compared to those not stimulated by LPS, indicating that LPS enhanced mitoxantrone extrusion in mDCs. This effect of LPS was completely inhibited by Ko143, a specific inhibitor of ABCG2 (Figure 1D). These results indicate that the functional ABCG2 is expressed in blood mDCs and its level is up-regulated by LPS.

### ABCG2 inhibitor suppresses the maturation of DCs

**Figure 2:**
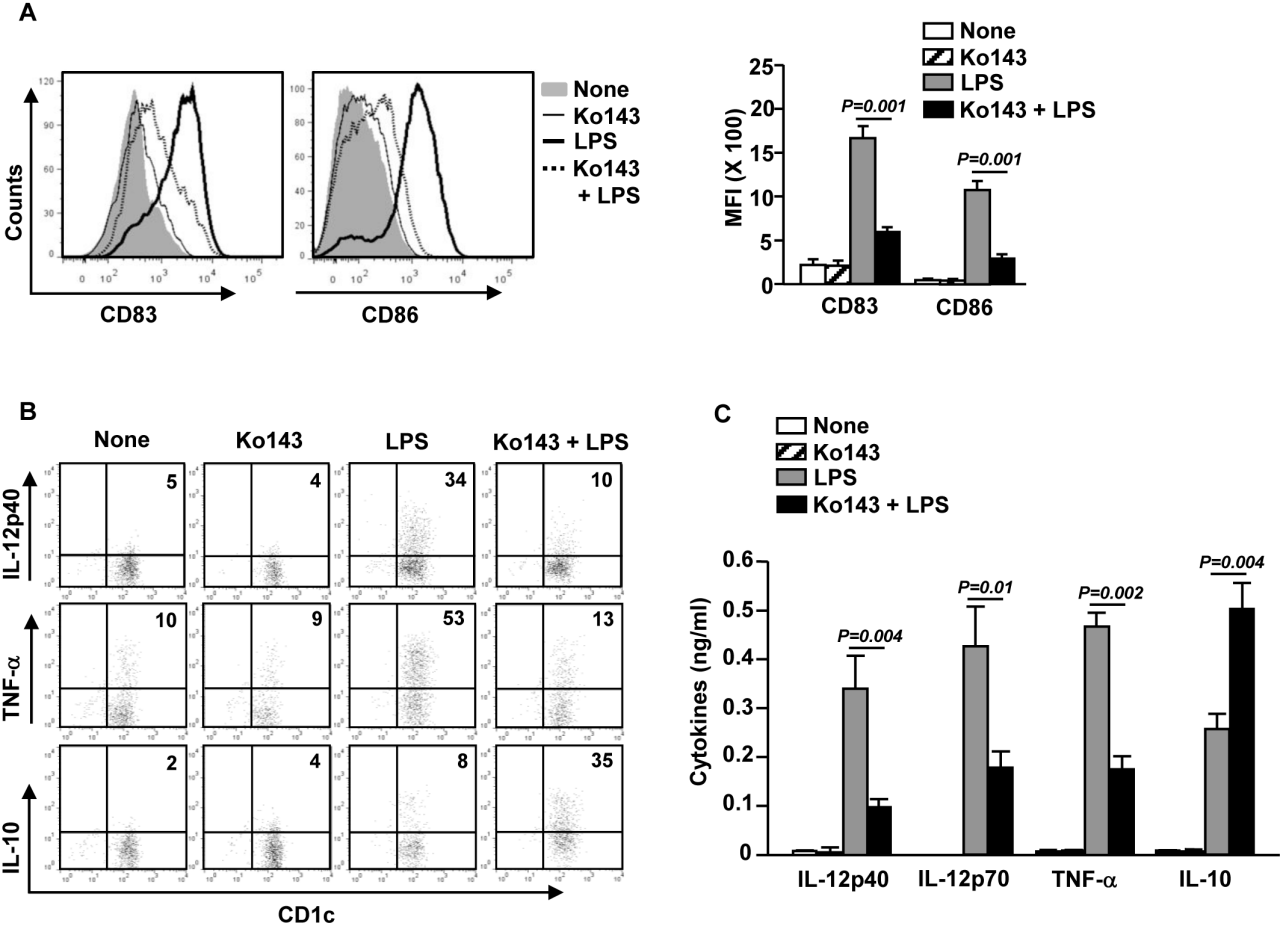
Ko143 suppresses LPS-induced mDC maturation. (A) Purified CD1c^+^ mDCs were pre-treated with Ko143 for 1 hour and incubated with or without LPS for 24 hours. CD83 and CD86 expression were analyzed by flow cytometry (left panel). MFI of CD83 and CD86 was shown (right panel). Data are representative or the average of analyses of 3 samples from 3 donors. (B) Intracellular IL-12p40, TNF-α and IL-10 production in purified CD1c^+^ mDCs were analyzed by flow cytometry. Data are representative or the average of analyses of 3 samples from 3 donors. (C) Concentrations of IL-12p40, IL-12p70, TNF-α and IL-10 in the culture medium of CD1c^+^ mDCs as measured by ELISA. Data are representative or the average of analyses of 6 samples from 6 donors.

Our observation that LPS up-regulated the expression of ABCG2 in DCs prompted us to examine whether inhibition of ABCG2 can affect DC maturation. We stimulated MDDCs with LPS in the presence or absence of Ko143. After 24 hours of culture, we noticed that the induction of CD83 and CD86 up-regulation by LPS was dramatically decreased by Ko143 (Figure S1C). It has been reported that human DC subsets can crosstalk and induce the activation of each other [22]. Therefore, we next examined whether purified CD1c^+^ mDCs, the major cell population in peripheral blood mDCs, can behave similarly as MDDCs. We pretreated CD1c^+^ mDCs with Ko143 before LPS treatment, and found that the up-regulation of CD83 and CD86 expression in CD1c^+^ mDCs by LPS was substantially abrogated in the presence of Ko143 (Figure 2A).

We next tested the effect of Ko143 on LPS-induced production of cytokines in DCs. Ko143 pretreatment significantly reduced LPS-induced interleukin-6 (IL-6), IL-12p40, IL-12p70 and TNF-α production in MDDCs, whereas it did not affect IL-1β production (Figure S1D). Furthermore, intracellular levels of IL-12p40 and TNF-α in CD1c^+^ mDCs induced by LPS were markedly decreased by Ko143 treatment (Figure 2B). Consistent with the intracellular cytokine levels in CD1c^+^ mDCs, LPS-induced secretion of IL-12p40, IL-12p70 and TNF-α was significantly decreased by Ko143 (Figure 2C). Importantly, Ko143 plus LPS treatment led to a marked increase in the production of IL-10, a critical anti-inflammatory cytokine, in MDDCs (Figure S1D) and CD1c^+^ mDCs (Figure 2B and C). Hence, inhibition of ABCG2 by Ko143 prevents LPS-induced DC maturation and converts LPS-stimulated pro-inflammatory DCs into IL-10-producing anti-inflammatory DCs.

### ABCG2 knockdown inhibits LPS-induced MDDC maturation

**Figure 3:**
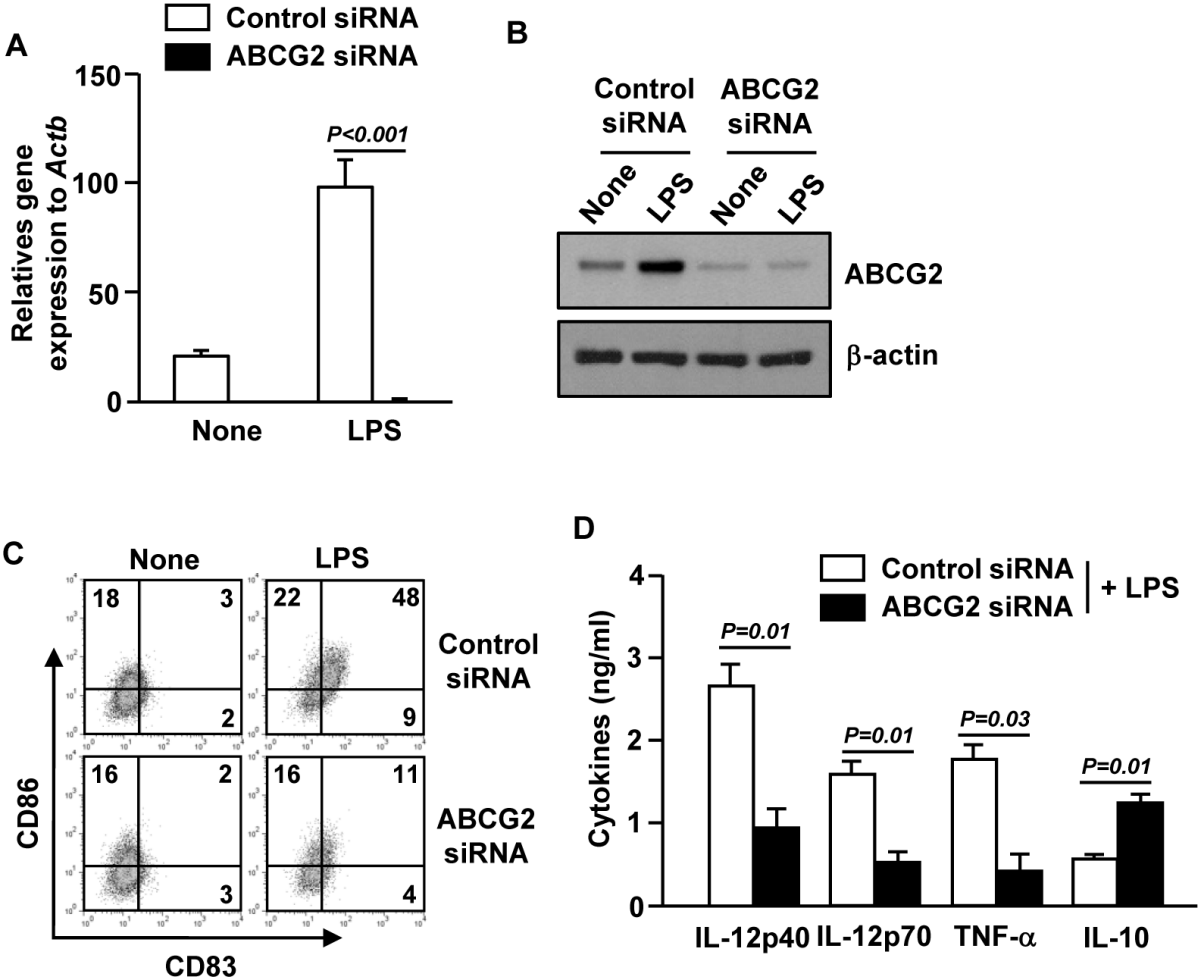
ABCG2 is required for LPS-induced DC maturation. MDDCs were transfected with ABCG2 siRNA or control siRNA for 24 hours and then stimulated with LPS for 24 hours. (A) mRNA levels of ABCG2 were measured by real time qPCR. (B) Protein levels of ABCG2 were subjected by western blotting using anti-ABCG2 Abs. (C) The expression levels of CD83 and CD86 were analyzed by flow cytometry. (D) Levels of IL-12p40, IL-12p70, TNF-α and IL-10 in the culture medium as measured by ELISA. All data are representative or the average of analyses of 4 samples from 4 donors for each group.

We next examined whether ABCG2 is required for LPS-induced DC maturation. For this experiment, immature MDDCs (iMDDCs) were used instead of CD1c^+^ mDCs because the numbers of purified CD1c^+^ mDCs were too low to perform knockdown experiments by siRNA. We analyzed the levels of the maturation markers on LPS-treated MDDCs in which ABCG2 was knocked down with siRNA. As shown in Figure 3A and B, efficient knockdown of ABCG2 in LPS-treated immature MDDCs (iMDDCs) was demonstrated by decreased mRNA and protein levels. LPS treatment could not induce up-regulation of CD83 and CD86 in iMDDCs in which ABCG2 was knocked down with siRNA (Figure 3C). Moreover, production of pro-inflammatory cytokines IL-12p40, IL-12p70 and TNF-α induced by LPS was also reduced by ABCG2 knockdown (Figure 3D). Similar to Ko143 treatment, Ko143 treatment, knockdown of ABCG2 in iMDDCs led to increased IL-10 production in response to LPS (Figure 3D). Hence, ABCG2 is required for LPS-induced DC maturation and silencing of ABCG2 expression promotes IL-10-production in these DCs.

### Ko143-induced anti-inflammatory mDCs promote expansion of Treg cells

**Figure 4:**
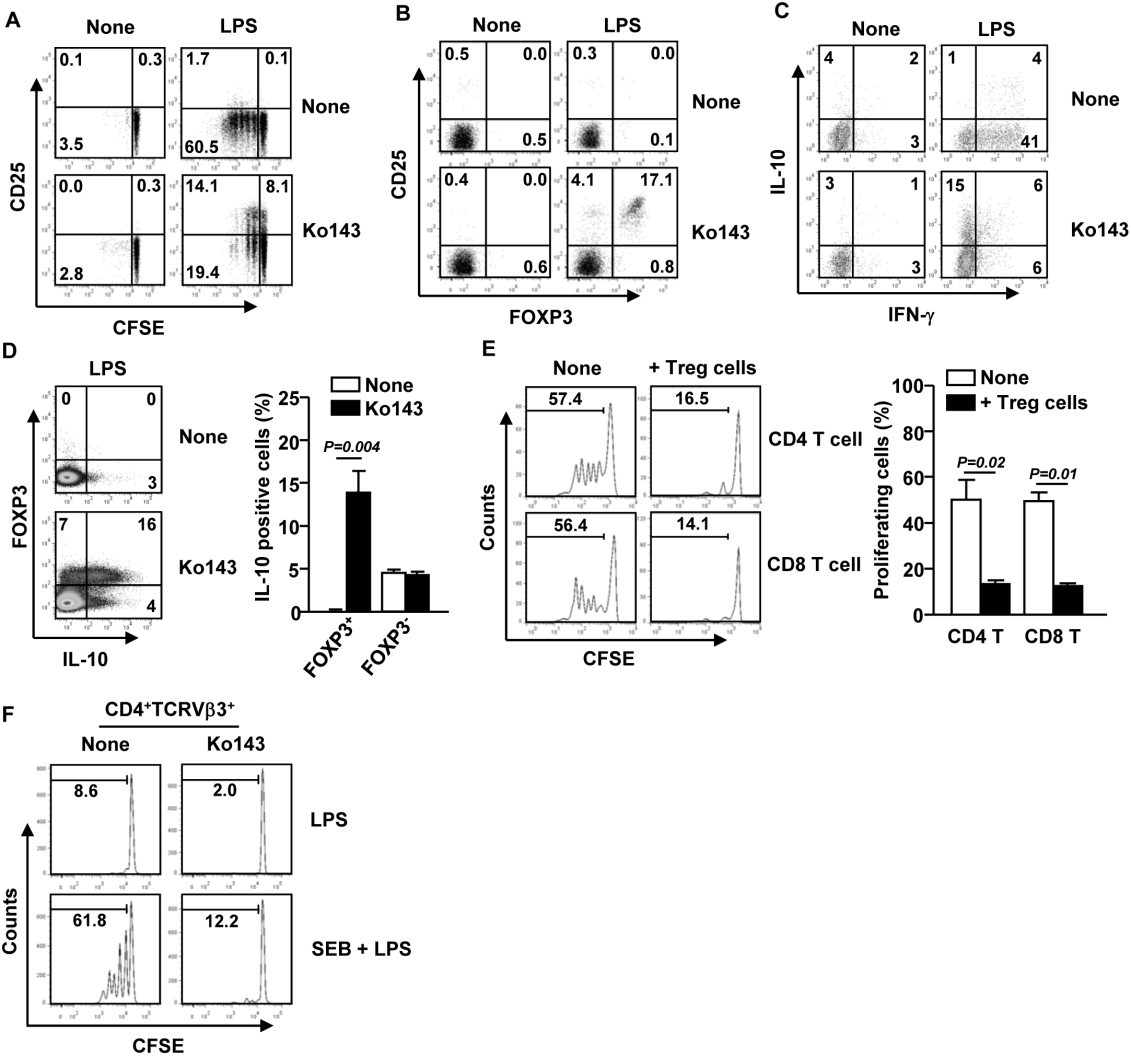
LPS plus Ko143-treated mDCs promote expansion of Treg cells. Ko143-, LPS- or LPS plus Ko143-treated CD1c^+^ mDCs were co-cultured with CFSE-labeled allogeneic CD4^+^ T cells (1×10^5^) in a 1∶10 ratio for 4 days. (A) Surface expression of CD25 and CFSE dilution was analyzed in CD4^+^ T cells by flow cytometry. (B) Flow cytometry of CD4, CD25 and FOXP3 expression was shown. Data are representative of analyses of 3 samples from 3 donors. (C) Flow cytometry of intracellular IL-10 and IFN-γ production in gated CD4^+^ T cells was shown. (D) Intracellular IL-10 expression in CD4^+^FOXP3^+^ T cells (left panel) and mean percentage of IL-10^+^FOXP3^+^ or IL-10^+^FOXP3^−^ cells (right panel) were shown. Data are representative or the average of analyses of 3 samples from 3 donors. (E) CFSE-labeled PBMCs were incubated with soluble anti-CD3 and CD28 Abs for 4 days in the presence or absence of CD25^+^ Treg cells. CFSE dilution was analyzed in CD4^+^CD25^−^ and CD8^+^ T cells (left panel) and mean percentage of proliferating cells (right panel) was shown. Data are representative or the average of analyses of 4 samples from 4 donors. (F) CD1c^+^ mDCs were cultured with LPS and CFSE-labeled syngenic CD4 T cells, in the presence or absence of Staphylococcal Enterotoxin B (SEB) and Ko143 for 4 days. CFSE dilution was analyzed in CD4^+^TCRVβ3^+^ T cells by flow cytometry. Data are representative of analyses of 3 samples from 3 donors.

Recent reports have demonstrated that IL-10-producing DCs induce Treg cell differentiation [23]. To determine whether CD1c^+^ mDCs treated with Ko143 and LPS can also drive Treg cell differentiation, we co-cultured LPS and Ko143-stimulated CD1c^+^ mDCs with allogeneic CD4 T cells. CD1c^+^ mDCs treated with the combination of LPS and Ko143 efficiently promoted the expansion of CD4^+^CD25^+^ T cells, whereas those treated with LPS alone did not (Figure 4A). Moreover, proliferation of CD4 T cells was reduced when cultured with LPS plus Ko143-treated mDCs compared to those cultured with LPS-stimulated mDCs, as measured by CFSE-labeling assay (Figure 4A). We next determined whether the expanded CD4^+^CD25^+^ T cells express FOXP3, the key transcription factor controlling Treg cell development and function. As shown in Figure 4B, a large proportion of CD25^+^FOXP3^+^ T cells were induced only by CD1c^+^ mDCs treated with LPS plus Ko143. In addition, CD1c^+^ mDCs treated with LPS plus Ko143 induced significant higher frequency of IL-10-producing CD4 T cells and lower frequency of IFN-γ-producing CD4 T cells compared to those treated with LPS alone (Figure 4C). Moreover, the majority of IL-10-producing T cells induced by LPS plus Ko143-treated CD1c^+^ mDCs were FOXP3^+^ cells, thus possessing the characteristics of Treg cells (Figure 4D).

To determine whether these Treg cells can functionally suppress T cell responses, we examined T cell proliferation with or without CD25^+^ Treg cells that were derived from co-culture with by LPS plus Ko143-treated CD1c^+^ mDCs. Purified CD4^+^CD25^+^ Treg cells were co-cultured with CFSE-labeled PBMCs in the presence of soluble anti-CD3 and anti-CD28 Abs. After 4 days of culture, the proliferation of CD4 and CD8 T cells among the PBMCs was significantly reduced by the presence of CD25^+^ Treg cells (Figure 4E). We next examined whether LPS plus Ko143-induced anti-inflammatory mDCs can inhibit antigen-specific T cell responses. CD1c^+^ mDCs were cultured with LPS and CFSE-labeled syngenic CD4 T cells, with or without Staphylococcal Enterotoxin B (SEB) and Ko143. After 4 days of culture, proliferation of SEB-specific TCRVβ3^+^ CD4 T cells was measured by flow cytometry. SEB induced a substantial increase in the proliferation of TCRVβ3^+^ CD4 T cells co-cultured with LPS-treated mDCs in the absence of Ko143 (Fig. 4F). Addition of Ko143 to this mixed culture system markedly reduced the proliferation of TCRVβ3^+^ CD4 T cells (Fig. 4F). In summary, LPS plus Ko143-induced anti-inflammatory mDCs promote the generation of IL-10-producing CD4^+^CD25^+^FOXP3^+^ T cells with phenotypic, genetic and functional traits of Treg cells. These data suggest that inhibition of ABCG2 during LPS stimulation of DCs promotes tolerogenic DC differentiation.

### IL-10 is essential for the generation of Treg cells promoted by LPS plus Ko143-induced tolerogenic mDCs

**Figure 5:**
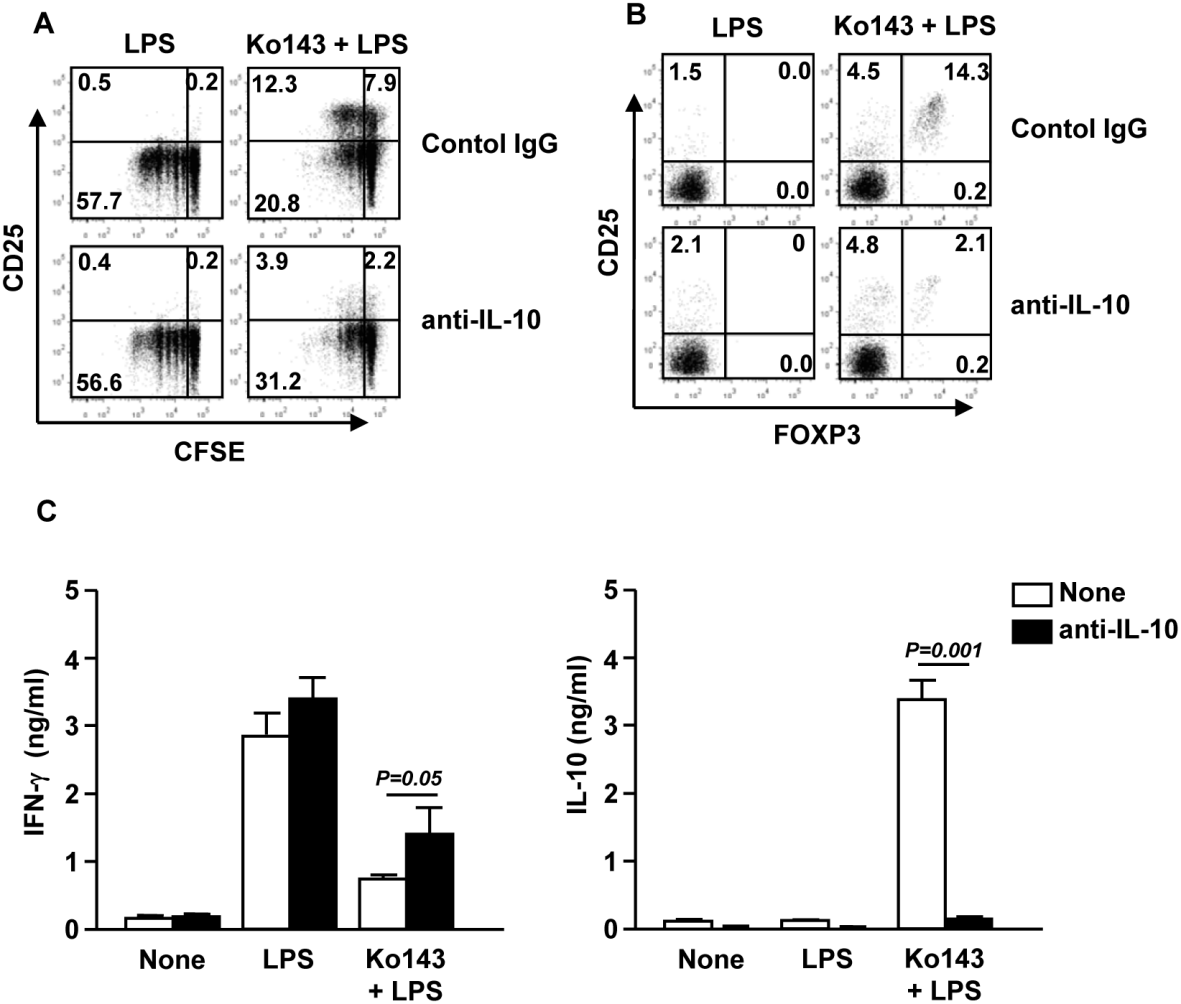
Generation of Treg cells induced by LPS plus Ko143-treated mDCs is dependent on IL-10. CD1c^+^ mDCs and CD4 T cells were co-cultured as described in Figure 4 in the presence of anti-IL-10 or control IgG Abs. (A) Expression of CD25 and CFSE dilution were analyzed in CD4^+^ T cells. (B) CD4, CD25 and FOXP3 expression was analyzed by flow cytometry. (C) IFN-γ (left panel) and IL-10 (right panel) concentrations in the culture medium were measured by ELISA. Data are representative or the average of analyses of 3 samples from 3 donors.

**Figure 6:**
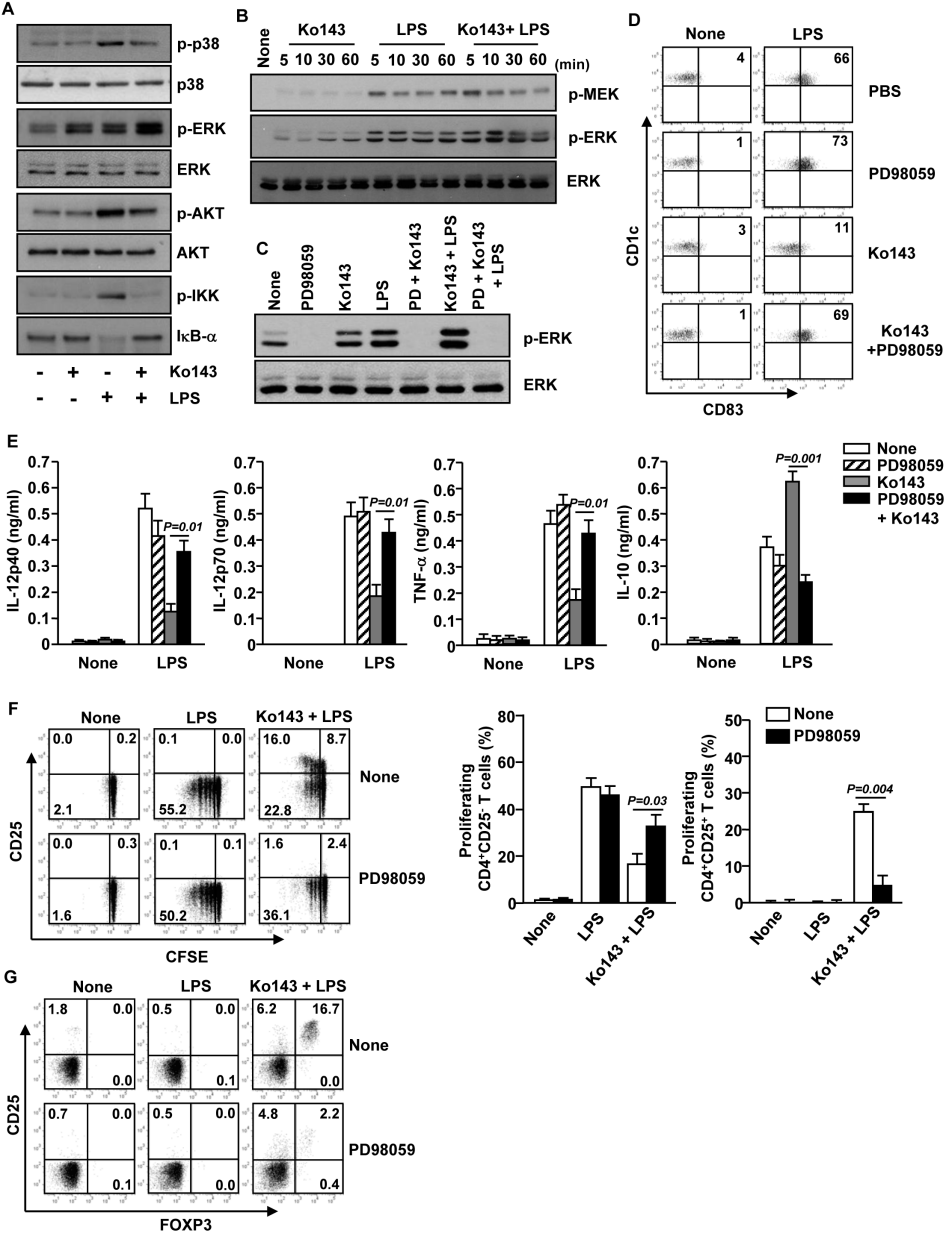
ERK pathway is essential for optimal induction of tolerogenic mDCs by LPS and Ko143. (A) MDDCs (1×10^6^) were pre-treated with or without Ko143 for 1 hour and then incubated with LPS for 10 minutes. The samples were then subjected to Western blotting using anti-phosphorylated p38 (p-p38), ERK (pERK), AKT (p-AKT) and IKK (p-IKK) Abs. (B) MDDCs were treated with Ko143, LPS or Ko143 plus LPS for indicated amount of time. Phosphorylation of MEK and ERK were subjected to Western blotting. (C) MDDCs were pre-incubated with PD98059 for 10 minutes and further cultured with Ko143, LPS or Ko143 plus LPS for 30 minutes. Phosphorylation of ERK was measured by Western blotting. (D) CD1c^+^ mDCs were stimulated with LPS together with Ko143 as described in Figure 2 in the presence or absence of PD98059. Flow cytometry of CD83 expression in CD1c^+^ mDCs is shown. (E) Concentrations of IL-12p40, IL-12p70, TNF-α and IL-10 in the culture medium. (F) CD1c^+^ mDCs were co-cultured with allergenic CD4 T cells as described in Figure 4. Expression of CD25 and CFSE dilution were analyzed in CD4^+^ T cells (left panel). Mean percentage of proliferating CD4^+^CD25^−^ cells (middle panel) or CD4^+^CD25^+^ cells (right panel) was shown. (G) CD4, CD25 and FOXP3 expression was analyzed by flow cytometry. Data in A–C are the representative of at least three independent experiments, and data in D–G are representative or the average of analyses of 4 samples from 4 donors for each group.

Previous studies have reported an essential role for IL-10 in the generation of Treg cells induced by tolerogenic DCs [24], [25]. To determine whether the promoting effect of LPS and Ko143-treated mDCs on Treg generation is dependent on IL-10, we prepared mDCs as described in Figure 4 and co-cultured them with allogeneic naïve CD4 T cells in the presence of a neutralizing anti-IL-10 Abs or its control IgG for 4 days. The results showed that the promoting effect of Ko143- and LPS-treated mDCs on CD25^+^ T cells was almost completely blocked by the anti-IL-10 Abs (Figure 5A). In contrast, anti-IL-10 Abs treatment caused a small increase in CD4^+^CD25^−^ T cell proliferation compare to control IgG treatment. Moreover, intranuclear staining showed that anti-IL-10 Abs markedly reduced FOXP3^+^ Treg cell generation induced by LPS plus Ko143-treated mDCs (Figure 6B). Furthermore, anti-IL-10 Abs treatment partially reversed the inhibition of T cell IFN-γ production and promotion of IL-10 production LPS plus Ko143-treated mDCs (Figure 5C). Hence, these data demonstrate that Treg cell generation induced by LPS plus Ko143-stimulated mDCs is dependent on IL-10.

### Induction of tolerogenic mDCs by Ko143 requires ERK phosphorylation

Mitogen-activated protein kinase (MAPK) and nuclear factor (NF)-κb have been shown to be required for the activation and maturation of DCs [26], [27]. We pretreated MDDCs with Ko143 for 1 hour and then incubated them with or without LPS for 10 minutes. LPS treatment up-regulated the phosphorylation of p38, AKT and IκB kinase (IKK) in MDDCs and the phosphorylation of these proteins was partially inhibited by Ko143 (Figure 6A). Interestingly, we found that Ko143 treatment alone up-regulated ERK phosphorylation, which was further enhanced by LPS (Figure 6A). In addition, MEK phosphorylation, a direct upstream event of ERK signaling pathway, was also increased by Ko143 treatment (Figure 6B). Furthermore, the combination of Ko143 and LPS had an additive effect on MEK and ERK phosphorylation (Figure 6B).

We next determined whether the generation of tolerogenic DCs induced by Ko143 plus LPS is dependent on ERK phosphorylation. We first analyzed the effect of PD98059, an ERK specific inhibitor, on Ko143, LPS or Ko143 plus LPS-induced phosphorylation of ERK. Pretreatment of MDDCs with PD98059 completely blocked ERK phosphorylation induced by Ko143, with or without LPS (Figure 6C). Moreover, in LPS-treated CD1c^+^ mDCs, the inhibition effect of Ko143 on CD83 expression was completely abolished by PD98059 (Figure 6D). Furthermore, Ko143-induced inhibition of IL-12p40, IL-12p70 and TNF-α production in LPS-stimulated mDCs were almost completely abrogated by ERK inhibition (Figure 6E). In addition, the induction of IL-10 production in DCs by Ko143 plus LPS treatment was also dramatically decreased by PD98059 (Figure 6E).

We next examined whether inhibition of tolerogenic mDC differentiation by PD98059 would prevent the induction of Treg expansion by these mDCs. The ability of Ko143 plus LPS-treated mDCs to stimulate CD4^+^CD25^+^ Treg generation was almost completely abrogated by PD98059 (Figure 6F). In contrast, proliferation of CD4^+^CD25^−^ T cells under the same condition was markedly increased by PD98059 (Figure 6F). Importantly, PD98059 treatment substantially inhibited FOXP3^+^ T cell expansion induced by Ko143 plus LPS-treated mDCs (Figure 6G). Taken together, these data indicate that Ko143-induced conversion of LPS-stimulated DCs into tolerogenic DCs requires ERK signaling pathways.

## Discussion

ABC transporters have immune modulating function in various immune effector cells, most notably DCs. Previous reports demonstrated that the ABC transporters P-glycoprotein, ABCG2, MRP1 and MRP4 are detected on human MDDCs and LCs [19], [28]–[30]. Moreover, ABCG2 expression can be regulated by various stimuli in MDDCs. PPARγ agonist positively regulates ABCG2 expression [21], whereas prostaglandin E2 (PGE2) and a cytokine cocktail of IL-1β, IL-6, and TNF-α negatively regulate ABCG2 expression in MDDCs [18]. In this study, we also found that human blood mDCs express ABCG2 and its expression is further enhanced by LPS stimulation. Moreover, the inhibition of ABCG2 during LPS-stimulation promotes IL-10-producing tolerogenic DCs. Similar with our observation, P-glycoprotein (Pgp; ABCB1), a molecule well known for its ability to transport of a broad spectrum of xenobiotics out of cells and thereby induce drug resistance, have important functions in DCs as blockade of ABCB1 inhibits differentiation, maturation and T cell-activating ability of MDDCs [20], [29], [31]. Although it has been shown that ABCG2 is required for MDDC and LC differentiation and migration [18], [32], [33], the inhibition of ABCG2 does not affect in DC maturation [18]. However, our results seem to be in disagreement with those of DC maturation results. The reasons for the discrepancies between their results and ours could be because of the different protocols used to DC maturation. Several protocols for DC maturation have been developed however each one has led to the different phenotypes and function. Especially, LPS-induced DC maturation has the different chemokine expression and T cell responses compare to cytokine cocktail-mediated DC maturation [34]. In this study, we demonstrate that the inhibition of ABCG2 prevents LPS-induced DC maturation, whereas it does not affect in cytokine cocktail-induced DC maturation [18]. Moreover, we showed that ABCG2 knockdown in MDDCs inhibits DC maturation induced by LPS. Furthermore, LPS up-regulates ABCG2 expression in DCs, whereas cytokine cocktail down-regulates ABCG2 expression in MDDCs [18]. Therefore, these different effects in ABCG2 expression may explain why ABCG2 is required in LPS-induced DC maturation but not in cytokine-induced DC maturation.

When ABC transporters are over-expressed in cancer cells, they pump out anti-cancer drugs from the cells, leading to decreased intracellular drug concentration and attenuated chemotherapeutic effect [35]. Therefore, inhibition of these transporters was expected to sensitize MDR cancer cells to chemotherapeutic drugs and enhances the efficacy of the treatment. However, most of the strategies used to surmount ABCG2–mediated MDR have had limited success [8], [36]. In this research, we demonstrated one possibility that treatment of ABCG2 inhibitor during chemotherapy may promote tolerogenic DC and Treg cell expansion. In cancer patients, immune-suppressive tumor microenvironment has been reported to promote tolerogenic DC differentiation and thereby promote the generation of Treg cells in the tumor and the lymph nodes [37], [38]. Various lines of evidence suggest that Treg cells critically contribute to immunological tolerance to cancer [39]. Moreover, expanded Treg cells not only suppress anti-cancer immunity but also directly promote cancer cell proliferation, invasion and metastasis [39], [40]. In the case of melanoma, cancer metastasis is accelerated by the induction of immunosuppression with injection of additional Treg cells [41]. An immunosuppressive state has been found in virtually all cancers and this state can be further reinforced by enhanced generation of tolerogenic DCs or Treg cells. Hence, our results suggest that using ABCG2 inhibitors to treat MDR cancers may promote the generation of tolerogenic DCs and expansion of Treg cells, thereby helping cancer growth and metastasis. This may be one of the reasons for the failure of MDR cancer therapy by ABCG2 inhibition. To address this question in humans, in future research, we will investigate whether the percentage and number of tolerogenic DCs and Treg cells are altered in MDR cancer patients receiving treatment with ABCG2 inhibitor and anti-cancer drugs.

NF-κB and MAPK signaling pathways are the most crucial signaling pathways for DC maturation [26], [42]. Inhibition of ABCG2 partially prevents LPS-induced phosphorylation of p38 and IKK in MDDCs, consistent with previous literature showing that p38 in MAPK pathway and IKK in NF-κB pathway are essential for DC maturation [26], [42]. Interestingly, we found that treatment of MDDCs with Ko143 alone induces ERK phosphorylation, which can be further increased by the presence of LPS. Moreover, the ERK pathway blocker PD98059 completely inhibits the induction of tolerogenic DCs by Ko143. ERK pathway is one of the major signaling pathways in DCs that are activated by toll-like receptor (TLR) ligands [26]. DCs treated with p38 inhibitor SB203580, but not ERK inhibitor PD98059, fail to up-regulate CD83 expression and pro-inflammatory cytokine production in response to LPS and TNF-α [26]. Furthermore, ERK pathway is required for IL-10 production from TLR agonist-treated DC [43]. Consistent with these reports, PD98059 does not affect maturation of DCs in response to LPS, but inhibits Ko143 plus LPS-induced IL-10 production from DCs. These data suggest that ERK signaling pathway may control IL-10 production in DCs in response to treatment with Ko143 plus LPS. Future studies are required to define the molecular mechanisms by which ERK controls the generation of tolerogenic DCs in response to Ko143 plus LPS.

During chemotherapy in patients with cancer, there is an increased risk of infections due to a low white blood cell count caused by the toxic effect of drugs on the bone marrow [44]. Bacterial infections are a major cause of morbidity and mortality in patients receiving chemotherapy for malignancy [44], [45]. In chemotherapy for MDR cancer, inhibition of ABCG2 is expected to enhance the efficacy of the anti-cancer drug treatment. However, we demonstrate here that ABCG2 inhibitor plus bacterial LPS can promote immune tolerance and immunosuppression. This finding suggests that using ABCG2 inhibitor for MDR cancer chemotherapy may increase the susceptibility of the patients to bacterial infections. Moreover, in cancer patients with ongoing bacterial infections, the effect of ABCG2 inhibitor can be particularly detrimental, as the combination of ABCG2 inhibitor and bacterial LPS can exacerbate the immunosuppressive state, worsening both the infection and the cancer. Further research will be required to test these possibilities in mouse models of bacterial infections and cancer.

In conclusion, we showed that LPS stimulation can up-regulate expression of ABCG2 in human blood mDCs. Inhibition of ABCG2 in LPS-stimulated mDCs transforms these cells into functional tolerogenic DCs. Hence, ABCG2 is essential for LPS-induced DC maturation and inhibition of ABCG2 suppresses DC maturation and promotes tolerogenic DC generation. Our work suggests that alteration of ABCG2 activity can affect the balance of immune activation versus tolerance and demonstrates the need for a deeper understanding of the function of ABCG2 and the effects of ABCG2 inhibitors on immune tolerance in order to develop better strategies to combat MDR cancer.

## Materials and Methods

### Ethics statement

This study was conducted according to the principles expressed in the Declaration of Helsinki. Elutriated PBDCs were obtained from healthy donors at the Shanghai Public Health Clinical Center. The Institutional Review Board at Shanghai Public Health Clinical Center approved this study (IRB number: 2012ZX09303013). Written informed consent was obtained from all volunteers.

### Chemicals and antibodies

Ko143 and LPS (*Eschericia coli* strain O111:B4) were obtained from Sigma Chemical Co (St. Louis, MO). Isotype control antibodies (Abs) (IgG1, IgG2a and IgG2b), anti-ABCG2-FITC (IgG2b, 5D3), anti-CD1c-APCcy7 (IgG1, L161), anti-CD4-PerCP/Cy5.5 (IgG2b, OKT4), anti-CD8-Pacific Blue (IgG1, HIT8a), anti-CD11c-APC (IgG1, 3.9), anti-CD123-PEcy7 (IgG1, 6H6), anti-CD83-FITC (IgG1, HB15e), anti-CD25-APC and -PEcy7 (IgG1, M-A251), anti-FOXP3-Pacific Blue and -Alexa Flour-488 (IgG1, 206D), anti-IL-10-PE (IgG2a, JES3-19F1) and anti-IFN-γ-Alexa Flour-488 (IgG1, 4S.B3) Abs were obtained from Biolegend; and neutralizing Ab against human IL-10 (IgG2a, JES3-19F1) was purchased from Biolegend. Abs against phosphorylated form of p38, AKT, ERK and phospho-IKK were obtained from Cell Signaling Technology (Beverly, MA).

### Isolation of PBDCs and CD1c^+^ mDCs

PBDCs were purified from the PBMCs by magnetically activated cell sorting using a blood DC isolation kit II from Miltenyi Biotec (Bergisch-Gladbach, Germany). Briefly, lineage positive cells were depleted by non-DC depletion cocktail (cD14, CD19) from PBMC, and then DC subsets were enriched by CD1c, CD304 and CD141 antibody. CD1c^+^ mDCs also purified form PBMC by CD1c DC isolation kit (Miltenyi Biotec, Bergisch-Gladbach, Germany). After depleted CD19^+^ cells from PBMC, CD1c^+^ DCs were enriched. The purity of CD11c^+^CD123^inter^ mDCs and CD11c^−^CD123^+^ pDCs were found to be >75% and CD1c^+^ mDCs were showed to be >95%. For some experiments, CD11c^+^CD123^inter^ mDCs were isolated by the cell shorter FACSAria II (Beckton Dickinson Franklin Lakes, NJ). Informed consent was obtained from all volunteers and the Institutional Review Board at Shanghai public health clinical center approved this study.

### Monocyte-derived DCs (MDDCs)

MDDCs were generated according to an established method with modifications [44]. Briefly, monocytes were purified from PBMCs by positive selection using a MACS column containing conjugated with Abs to CD14 (Miltenyi Biotec, Bergisch Gladbach, Germany). The purity of isolated CD14^+^ monocytes was >95% as determined by flow cytometry. The monocytes (1×10^6^) were incubated with 1000 U/ml recombinant human (rh) granulocyte and macrophage colony-stimulating factor (GM-CSF) plus 800 U/ml rh IL-4 in DC culture medium (RPMI-1640 medium, 10% autologous serum, 100 U/ml penicillin/streptomycin) for 6 days. The differentiation of immature MDDCs (iMDDCs) was monitored by the up-regulation of CD1a expression and down-regulation of CD14 expression.

### Efflux assay

The isolated mDCs (2×10^5^) were suspended in phenol red-free HPMI medium containing 20 µM mitoxantrone with or without Ko143 and incubated for 30 minutes at 37°C. Cells were washed twice in ice-cold PBS, re-suspended in HPMI medium and allowed to efflux for 90 minutes at 37°C. After washed twice in ice-cold PBS, Cells were suspended and analyzed on a FACSAria II (Beckton Dickinson Franklin Lakes, NJ).

### siRNA transfection of ABCG2

The siRNA sequence used for the targeted silencing of ABCG2 was obtained by Santa Cruz Biotechnology. iMDDCs (10^6^) were left unmanipulated, transfected with siRNA Transfection Reagent System (Santa Cruz, CA), with 200 nM nonsilencing siRNA or with 200 nM ABCG2 siRNA for 24 hours. The efficient of siRNA tranfection was determined by real-time RCR and western blotting.

### Intracellular and intranuclear staining

Cells were stimulated with phorbol 12-myristate 13-acetate (50 ng/ml) and ionomycin (1 µM; both from Calbiochem, La Jolla, CA) for 4 hours, with the addition of monensin solution (Biolegend, San Diego, CA) during the final 2 hours. For intracellular cytokine staining, cells were stained for surface molecules first, then fixed and permeabilized with Cytofix/Cytoperm buffer (eBioscience, San Diego, CA) and subsequently incubated with indicated anti-cytokine antibodies in Perm/Wash buffer (eBioscience, San Diego, CA) for 30 minutes. Intra nuclear FOXP3 staining was performed by using a FOXP3 Fixation/Permeabilization kit (eBioscience, San Diego, CA) following the manufacturer’s instruction. Control staining with isotype control IgGs was performed in all the experiments.

### ELISA assay

The IL-1β, IL-6, IL-10, IL-12p40, IL-12p70 and TNF-α concentrations in the cell cultured media were measured in triplicate using standard ELISA kits (Biolegend, San Diego, CA), with standard cytokine preparations being used as the internal controls.

### T cell proliferation assay

Isolated naïve CD4 T cells were labeled with 5 µM carboxyfluorescein diacetate succinimidyl ester (CFSE) (Invitrogen, Carlsbad, CA) then co-cultured with activated mDCs. The cell mixtures were then cultured for 4 days at 37°C in RPMI 1640 medium under 5% CO_2_. The cells were stained with surface anti-CD4 and CD25 then analyzed by FACS Aria II for CFSE dilution in proliferating cells.

### Suppression assay by Treg cells

CD4^+^CD25^+^ Treg cells were sorted by FACS Aria II. PBMCs in RPMI 1640 containing 10% autologous serum plus 100 unit/ml penicillin, 100 µg/ml streptomycin, and 2 mM glutamine were incubated with 2 µM CFSE at 37°C for 10 min. Cells were then washed three times to remove unbound CFSE, resuspended in fresh medium. A total of 0.1×10^6^ labeled cells were activated with soluble anti-CD3/CD28 antibodies and cultured with or without 0.05×10^6^ CD4^+^CD25^+^ Treg cells. After 4 days, the cells were stained with surface anti-CD4, CD8 and CD25 Abs then analyzed CD4^+^CD25^−^ or CD8^+^ cells for CFSE dilution in proliferating cells by FACS Aria II.

### SEB specific T cell proliferation assay

CD1c^+^ mDCs were incubated with/without SEB, LPS, with/without ABCG, and together with CFSE-labeled syngenic CD4 T cells. After 4 days of culture, the cells were stained with surface anti-CD4 and anti-TCRVβ3 Abs and analyzed for CFSE dilution in TCRVβ3^+^ cells on a FACS Aria II.

### Western blotting

Cultured MDDCs (1×10^6^) were suspended in a lysis buffer containing 20 mM Tris-HCl (pH 7.4), 50 mM NaCl, 1% Triton X-100, and protease inhibitors. The lysates were then subjected to sodium dodecyl sulfate-polyacrylamide (SDS) gel electrophoresis, after which they were transferred to nitrocellulose membranes that were blocked for 1 hour at 25°C with a blocking buffer and then incubated with primary Abs in blocking buffer overnight at 4°C. The samples were treated with secondary Abs in a blocking buffer. Signals were detected by ECL chemiluminescence (Amersham, Uppsala, Sweden).

### Real time qPCR

Total RNA was reverse-transcribed into cDNA using Oligo (dT) and Superscript III (Invitrogen) or M-MLV reverse transcriptase (Promega, Madison, WI). The cDNA was subjected to real-time PCR amplification (Qiagen, Venlo, Limburg) for 40 cycles with annealing and extension temperature at 60°C, on a Light Cycler 480 Reat-Time PCR System (Roche, Swiss). The sequences of the primers used for ABCG2 detection were 5′-TTTCCAAGCGTTCATTCAAAAA-3′ (forward primer), 5′-TACGACTGTGACAATGATCTGAGC-3′ (reverse primer).

### Statistical analysis

Results are expressed as the mean ± standard error of the mean (SEM). The statistical significance of differences between experimental groups was calculated using analysis of variance with a Bonferroni post-test or an unpaired Student’s *t*-test. All p-values <0.05 were considered significant.

## Supporting Information

Figure S1 **Ko143 suppresses LPS-induced MDDC maturation.** (A) MDDCs were cultured with or without LPS for 24 hours. Surface expression levels of ABCG2 were measured by flow cytometry. (B) Real-time PCR analysis of ABCG2 gene expression, presented relative to that of β-actin. Data are representative of or the average of three independent samples. (C) MDDCs were pre-incubated with Ko143 for 1 hour and cultured with or without LPS for 24 hours. Surface expression levels of CD83 and CD86 were measured by flow cytometry. Cytokine concentrations in cultured medium were measured by ELISA. Data represent the mean ± SEM of three independent experiments.
(PDF)
