# Supplementary figures and images for: Inhibition of Breast Cancer Resistance Protein (ABCG2) in Human Myeloid Dendritic Cells Induces Potent Tolerogenic Functions during LPS Stimulation
Source: PLoS One. 2014 Aug 11;9(8):e104753. doi: 10.1371/journal.pone.0104753 (PMC4128747; doi:10.1371/journal.pone.0104753)

Figure S1

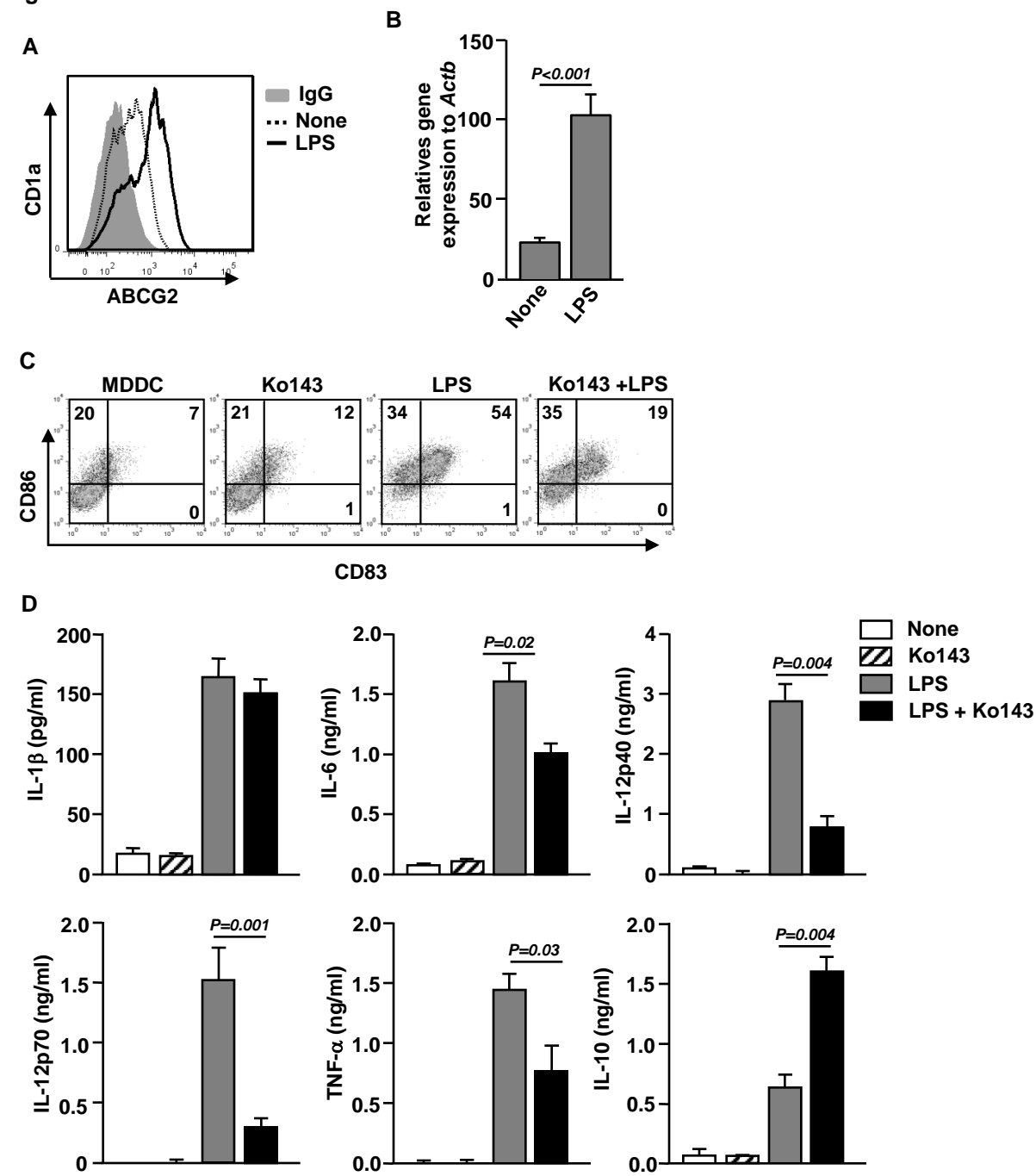

Supplement: Figure S1 — Ko143 suppresses LPS-induced MDDC maturation. (A) MDDCs were cultured with or without LPS for 24 hours. Surface expression levels of ABCG2 were measured by flow cytometry. (B) Real-time PCR analysis of ABCG2 gene expression, presented relative to that of β-actin. Data are representative of or the average of three independent samples. (C) MDDCs were pre-incubated with Ko143 for 1 hour and cultured with or without LPS for 24 hours. Surface expression levels of CD83 and CD86 were measured by flow cytometry. Cytokine concentrations in cultured medium were measured by ELISA. Data represent the mean ± SEM of three independent experiments. (PDF) [file pone.0104753.s001.pdf]
